# Farnesyl-transferase inhibitor R115,777 enhances tamoxifen inhibition of MCF-7 cell growth through estrogen receptor dependent and independent pathways

**DOI:** 10.1186/bcr1357

**Published:** 2005-11-21

**Authors:** Florence Dalenc, Claire Giamarchi, Mélissa Petit, Marc Poirot, Gilles Favre, Jean-Charles Faye

**Affiliations:** 1 INSERM U563, CPTP, Département Innovation Thérapeutique et Oncologie Moléculaire, Toulouse F-31052, France; 2 Institut Claudius Regaud, 42 rue du pont Saint Pierre, 31052 Toulouse Cédex, France; 3 Université Paul Sabatier, Toulouse F-31062, France

## Abstract

**Introduction:**

We have previously shown that FTI-277, a farnesyl transferase inhibitor (FTI), enhances the efficacy of tamoxifen (Tam) in inhibiting the proliferation of the estrogen dependent MCF-7 cell line. As the cellular response to Tam is the result of an inhibition of both estrogen receptor-dependent and -independent pathways, we have used the estrogen receptor selective anti-estrogen ICI182,780 and N-pyrrolidine(-phenylmethyl-phenoxy)-ethanamine-HCl (PBPE), a selective ligand of anti-estrogen binding site (AEBS), to dissect out the mechanism(s) associated with the observed additivity resulting from combination treatment with FTI-277 and Tam. Moreover, for these studies, FTI-277 has been replaced by R115,777, a FTI currently in phase III clinical trials.

**Methods:**

The quantitative sulphorhodamine B (SRB) colorimetric assay was used to determine the growth inhibitory effect of agents on MCF-7 cells. Dose response interactions between R115,777-Tam, R115,777-ICI182,780 and R115,777-PBPE were evaluated, at the IC_50 _point, using the isobologram method. Apoptotic cell death (DNA fragmentation, nucleus condensation and cytokeratin 18 cleavage) and inhibition of the mevalonate pathway (western blot) were also determined.

**Results:**

Combinations of the specific FTI R115,777 with either ICI182,780 or PBPE exhibit a synergistic effect on MCF-7 cell growth inhibition, while its combination with Tam is additive, as previously reported for FTI-277. Apoptosis is detected after treatment with combinations of R115,777 with either Tam or PBPE but not with ICI182,780, suggesting that each combination inhibits cell proliferation by different mechanisms. Even though the ER pathway has not yet been deciphered, it is shown here that the AEBS pathway is able to interfere with the mevalonate pathway at the level of protein farnesylation.

**Conclusion:**

Overall, this work reveals that combinations of R115,777 with either selective ER ligands or a selective AEBS ligand are able to induce large increases in their anti-proliferative activities on MCF-7 cells. Moreover, these results suggest that it may be of definite interest to evaluate combinations of R115,777 with different anti-estrogens in the treatment of ER positive breast tumours. Based on these experimental data, such combinations may prove beneficial in different clinical scenarios or when used in specific sequences; studying the combination of R115,777 with ICI182,780 for early treatment and reserving combinations with either Tam or a selective AEBS ligand, such as BMS-217380-01, for more resistant disease.

## Introduction

Tamoxifen (Tam) remains the most frequently prescribed agent for the treatment of hormone responsive breast cancer. Efficacy for Tam has been demonstrated in the treatment of all stages of breast cancer and it is also used as prevention therapy for women at high risk of developing breast cancer. Although Tam is effective initially in many patients, a major obstacle to its long-term use is tumour resistance. To resolve this problem, we decided to evaluate the effects of combining Tam with a farnesyl transferase inhibitor (FTI). Although the role of combination therapy versus sequential therapies with single agents remains controversial in the treatment of breast cancer, combination therapies using two or more antitumour drugs with differing mechanisms of action have generally proved more effective than single-drug therapies. In this basis, combinations of FTIs with a variety of commonly used anticancer agents have been tested on human tumours [1]. The FTI R115,777 represents one of the first-in-class inhibitors to enter the clinic [2]. Clinical trials have shown that this compound has an acceptable safety profile, allowing the administration of biologically active doses. Phase II clinical trials in patients with metastatic breast carcinoma have shown that R115,777 has reproducible single-agent activity [3]. In this present study, the activity of combined treatment with Tam and R115,777 was investigated using a mammary cancer cell line (MCF-7), and the results are compared with those from our previous studies using FTI-277 (an experimental FTI not in clinical usage) to evaluate the potential value of this combined therapy in the treatment of advanced breast cancer.

The antitumour effect of Tam is believed to be due to a combination of hormone receptor dependent (estrogen receptor (ER)-mediated with two receptor subtypes, ERα and ERβ) and independent (non-ER-mediated) mechanisms [4]. The signalling proteins in the non-ER-mediated pathways [5] include interactions with microsomal anti-estrogen-binding sites (AEBS) that are high affinity binding sites for Tam [6,7]. AEBS are different from ERs because they have no affinity for 17β-estradiol or steroidal anti-estrogens, and we recently reported that these sites consist of several proteins involved in cholesterol metabolism [8]. Previous reports have suggested that Tam inhibits MCF-7 cell proliferation [9] by binding to both ERs and AEBS [7,10] and can induce apoptotic cell death, both *in vitro *[11] and *in vivo *[12].

In parallel, FTIs inhibit the growth of a broad variety of human tumour cells *in vitro *and studies to date have not identified any cellular characteristics that predict the antitumour efficacy of this class of agent. *In vitro*, however, FTI treatment of tumour cells has been associated with activation of apoptotic pathways [13].

We have previously shown the involvement of pocket proteins [14] and genomic ER effects [15] in the additive efficacy of a combination of Tam and FTI277. To dissect out that portion of the Tam effect associated with the 'AEBS pathway', we compare here the effects of combining R115,777 with a selective AEBS ligand (N-pyrrolidine(-phenylmethyl-phenoxy)-ethanamine-HCl (PBPE)) [10] or with a selective estrogen receptor ligand (ICI182,780) on MCF-7 cell proliferation. The effects of such treatments on the induction of cellular apoptosis have been evaluated by monitoring cell cycle alterations and caspase involvement. The contribution of ERs to apoptosis induction has also been determined by comparing the effects of combined treatments with either the pure anti-estrogen ICI182,780 or the AEBS selective ligand PBPE.

## Materials and methods

### Cell lines

The human adenocarcinoma breast cell line MCF-7 was obtained from the American Tissue Culture Collection (Manassas, VA, USA). MCF-7 cells were grown routinely in RPMI 1640 medium supplemented with 5% fetal bovine serum (Gibco BRL, Life Technologies, Cergy Pontoise, France) and 2 mM L-glutamine. The cells were incubated at 37°C in a humidified 5% CO_2 _incubator.

For all experiments, the cells were treated with R115,777 (Janssen Research Foundation, Janssen Pharmaceutica, L.P., Titusville, NJ 08560, USA), Tam (Sigma-Aldrich S.a.r.l. St Quentin Fallavier, France), ICI182,780 (Tocris Bioscience, Ellisville, Missouri 63021 USA), PBPE (synthesized in our laboratory) [16] or vehicle, and the medium was changed every 2 days. R115,777, Tam, ICI182,780 and PBPE were all dissolved in ethanol and then diluted 10^3^-fold directly into the culture medium.

### Sulphorhodamine B assay for proliferation

The quantitative sulphorhodamine B (SRB) colorimetric assay [17] was used to determine the growth inhibitory effect of drugs on MCF-7 cells. Cells were seeded at 3,500 per well in 96-well plates and grown for 24 h. The cells were then treated with increasing concentrations of compounds and grown for a further 5 days. The medium was changed after 2 days. At the end of the incubation, cells were fixed with 50% trichloracetic acid (1 h at 4°C), stained for 30 minutes at room temperature with 50 μl of a 0.4% w/v SRB solution (Sigma) in 1% acetic acid. SRB was then removed and cells were quickly rinsed four times with 1% acetic acid. After air-drying, protein-bound dye was dissolved in 150 μl of 10 mM unbuffered Tris base (pH 10.5) for 5 minutes on a gyratory shaker. The pink SRB was quantified by measuring the optical density at 540 nm. For each condition, average cell density and the standard deviation were calculated from the data of six wells.

### Isobologram analysis

Dose response interactions between the following combinations: R115,777-Tam, R115,777-ICI182,780, R115,777-PBPE at the IC_50 _(inhibitory concentration 50%) point were evaluated by the isobologram method of Steel and Peckham [18]. When the data points of the drug combination fall within the area surrounded by two lines (envelope of additivity), the combination is regarded as additive. When the data points of the drug combination fall to the left of the envelope (i.e., the combined effect is caused by lower doses of the two agents than predicted), the combination is regarded as having a supra-additive effect (synergism). Finally, when the data points fall to the right of the envelope, the combination is regarded as having a protective effect.

To determine the envelope of additivity, the two dose-response curves of R115,777 with either Tam, ICI182,780, or PBPE alone were plotted according to bi-exponential equations. Based on these data, two isoeffect curves were constructed for each association. In all cases, an incremental effect was produced by R115,777 for any selected dose of Tam, ICI182,780, or PBPE.

#### Mode I line

The addition was calculated by taking the increment in dose starting from 0 that produced a log cell density that summated to the IC_50 _(hetero-addition where there would be no influence of drug treatment on R115,777 action). If the agents are acting additively through independent mechanisms, the combined data points would lie near this mode I line.

#### Mode II line

The addition was calculated by taking the increment in dose starting from the point on the dose-response curve of Tam, ICI182,780 or PBPE that produces a log cell density that summated to the IC_50 _(iso-addition where drug treatment would modify the basal state of the cells before R115,777 action). If the agents are acting additively through a similar mechanism, the combined data points would lie near this mode II line.

### Cell cycle analysis

For each condition, 1.5 × 10^5 ^cells were seeded into 60 mm diameter dishes and treated as described above. Following treatment, cells were collected by trypsinization, washed twice and resuspended in 500 μl ice cold PBS. The cells were then fixed in 1.5 ml ice cold absolute ethanol for 30 minutes at 4°C, washed twice in PBS and stained with propidium iodide (100 μg/ml RNase A in PBS, 0.25% Tween 20 and 50 μg/ml propidium iodide) for 1 h at 37°C. DNA content was determined by flow cytometry on a FACS Calibur (Beckton Dickinson and Co., Meglan, France). Data were collected from 10,000 cells. The percentage of apoptotic cells was calculated by dividing the number of cells displaying red fluorescence lower than the G0–G1 diploid peak by the total number of cells collected times 100.

### DAPI staining

Cells were grown on glass coverslips in 60 mm Petri dishes, washed once with PBS, fixed in PBS/3.7% formaldehyde for 15 minutes and washed twice with PBS. The coverslips were then mounted on glass slides with Vectashield mounting medium with 4'-6-diamidino-2-phenylindole (DAPI; AbCys 75010 Paris, France). In each experiment, a minimum of 200 nuclei were quantified using the ImageQuant software (Molecular Dynamics Inc., Sunnyvale, CA, USA).

### Detection of caspase cleavage by flow cytometry

Floating and adherent cells were combined and fixed in methanol at -20°C. After 30 minutes, the cells were washed twice with PBS containing 0.1% Tween 20. Non-specific binding was blocked with PBS containing 1% bovine serum albumin and 0.1% Tween 20 at room temperature. After 10 minutes, the blocking buffer was removed and the cells were incubated in 100 μl M30 CytoDeath antibody (1:100; Boehringer Ingelheim, France) at room temperature for 60 minutes. To visualize M30 CytoDeath antibody, a FITC conjugated second antibody was used (Boehringer) and the FITC signal was evaluated by flow cytometry. The results are expressed as an index of specific fluorescence (median fluorescence intensity - median fluorescence intensity of untreated cells/median fluorescence intensity of untreated cells × 100).

### Detection of caspase cleavage by immunocytochemistry

Floating and adherent cells are represented in cytospin preparations. Cells were fixed in absolute ethanol/acetic acid (99:1) for 1 minute. The staining was performed by a Techmate Horizon™ slide processor using a two-step peroxidase-conjugated polymer backbone visualisation system (EnVision™, DAKO, Glostrup, Denmark) according to the manufacturer's protocol. The chromogenic substrate was DAB (3,3'-diaminobenzidine). The primary antibody used was the M30 CytoDeath antibody (Boehringer). Negative controls were performed by omission of the primary antibody. All determinations were performed with at least 400 cells being quantified with the ImageQuant software for each experimental condition.

### Western-blot analyses

At the completion of the experiments, MCF-7 cell monolayers were washed with ice-cold PBS (Biowittaker Walkersville, MD, USA) and were then scraped into 100 μl of ice-cold lysis buffer: 50 mM HEPES, pH 7.5, 150 mM NaCl, 10% (v/v) glycerol, 1% Triton X-100, 1.5 mM MgCl_2_, 1 mM EGTA, 1 mM dithiothreitol and protease cocktail inhibitor (Sigma). The lysates were then placed on ice, vortexed vigorously at intervals over 10 minutes, centrifuged at 15,000 g for 10 minutes at 4°C and the supernatants stored at -80°C.

Equal amounts of total protein (5 μg) were submitted to 12.5% SDS-PAGE and then transferred to PVDF membranes. Proteins were visualized using the ECL+ detection system (Amersham Biosciences Europe GmbH,(Succursale France, F-91898 Orsay Cedex, France)) after incubation (overnight at 4°C for primary and 1 h at room temperature for secondary antibodies) using the primary antibody HDJ2 from Santa Cruz Biotechnology Inc. (Santa Cruz, CA, USA) and the secondary antibody anti-mouse horseradish peroxidase.

Protein abundance was quantified by analysis of autoradiographs. Relative band intensities were quantified by densitometric analysis (Molecular Dynamics, Sunnyvale, CA, USA). Quantification of protein levels by this method was linear over the range of protein concentrations analysed and exposure times employed in these studies.

### Cellular levels of [^3^H]tamoxifen

Cells were seeded at 180,000 per dish in 60 mm dishes and incubated with Tam and/or R115,777 as described above in the presence of [^3^H]tamoxifen (84 Ci/mmol; Amersham). Following such treatment, the cells were washed with PBS, collected by trypsinisation and counted for radioactivity in ready Emulsifer-safe scintillant (PerkinElmer Boston, MA 02118, USA).

## Results and discussion

### Effects of R115,777 when combined with different anti-estrogens on MCF-7 cell proliferation

**Figure 1:**
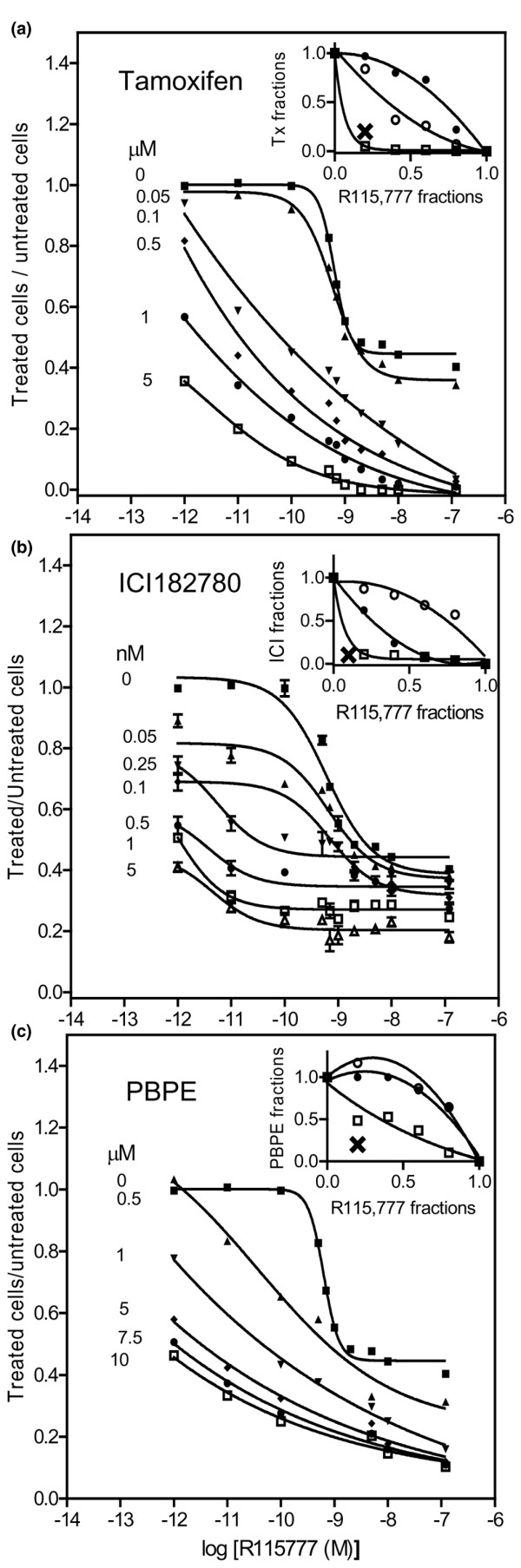
Effects of combining anti-estrogens and R115,777 on the inhibition of MCF-7 cell proliferation. Studies were performed using 1.5 × 10^5 ^cells in 60 mm dishes incubated for 5 days with increasing concentrations of **(a) **R115,777 and tamoxifen, **(b) **R115,777 and ICI182,780, or **(c) **R115,777 and PBPE. Dose response interactions between these anti-estrogens and R115,777 were evaluated using the isobologram method. The isoeffect curves were constructed, at the IC_50 _point, according to the Materials and methods [18]. X symbolizes the data point obtained with a combination of the two agents giving 50% inhibition. **(a) **When X falls within the envelope of additivity, the combination is considered as having an additive effect. **(b,c) **When X falls to the left of the envelope the combination is considered as having a supra-additive effect (synergism). PBPE, N-pyrrolidine(-phenylmethyl-phenoxy)-ethanamine-HCl.

We assessed the ability of R115,777 alone and in combination with anti-estrogens to inhibit the proliferation of MCF-7 cells. Cells were incubated for 5 days with incrementally increasing concentrations of R115,777 together with either Tam, ICI182,780 (pure anti-estrogen) or PBPE (selective AEBS ligand). For each compound, we first plotted individual dose response curves from which the following IC_50 _values were derived: 5.9 nM (R115,777), 4.4 μM (Tam), 0.52 nM (ICI182,780) and 8.8 μM (PBPE). Analyses of cell proliferation curves (Fig. 1a–c) showed strong inhibitory effects at low concentrations of R115,777 when associated with each of the three anti-estrogens and there was a suggestion of synergy for each of the combined pairs. To construct isobolograms according to the method described by Steel and Peckham [18] we carried out another set of experiments using combinations of the two drugs at concentrations resulting in 50% cell growth inhibition. Additive effects close to synergistic were observed between Tam and R115,777 (Fig. 1a, insert), confirming our earlier results using another FTI (FTI-277) from a different chemical class in association with Tam [14]. Although it can be argued that there is only a tenuous difference between additivity and synergy for this combination of an FTI with Tam, and it is accepted that this type of analysis is not really precise enough to definitively establish additivity between two agents, the methodology does identify clear additivity with two different FTIs with diverse molecular structures. To extend this observation to the evaluation of other anti-estrogens further, isobolograms were constructed. Isobologram analyses (Fig. 1b,c, inserts) revealed a synergistic inhibition of MCF-7 growth with combinations of R115,777 with both ICI182,780 or PBPE. Because the main high affinity targets of Tam are ERs and AEBS [5,6,8,19,20] and because of the synergistic effects between R115,777 and the ER ligand, results in agreement with data from Ellis *et al*. [21], together with the additive or synergistic effects observed with Tam, we had expected a negative effect with the combination that includes the selective AEBS ligand. Surprisingly though (Fig. 1c), PBPE also synergizes with R115,777, suggesting that cross-talk between FTIs and Tam is likely to occur via at least two different pathways.

To determine at what level this cross-talk occurs, we analysed the combined effects of R115,777 and Tam on various markers of cellular apoptosis. The rationale for this study was provided by an earlier publication by Ellis *et al*. [21] who proposed that hydroxy-tamoxifen and ICI182,780 (two selective ligands) worked synergistically with FTI-277 in the induction of apoptotic cell death in ER positive breast tumour lines.

### R115,777 enhances the apoptotic effect of tamoxifen

**Figure 2:**
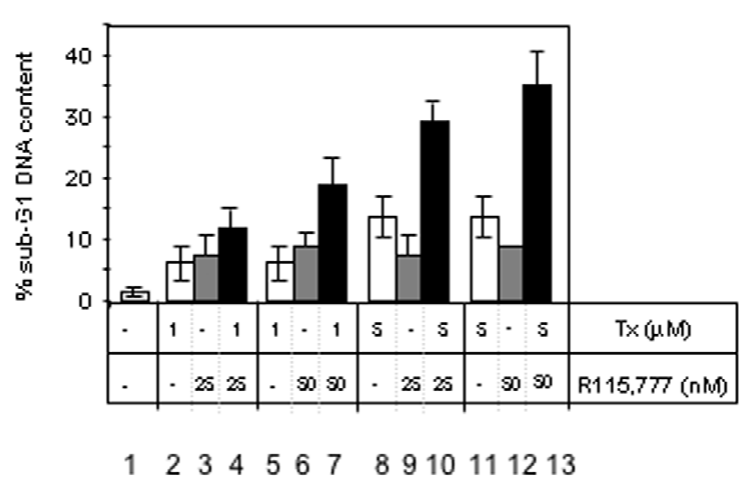
DNA fragmentation was determined using flow cytometric analysis. MCF-7 cells were incubated for 5 days with tamoxifen (Tx) and R115,777. Floating and adherent cells were harvested, fixed and their DNA was stained using propidium iodide. Data are shown as the percentage of cells with a sub-G1 DNA content over the total number of cells. Each point is the mean value from 6 × 10^4 ^cells, and data are expressed as the mean of three independent experiments. Error bars indicate the standard error of the mean.

**Figure 3:**
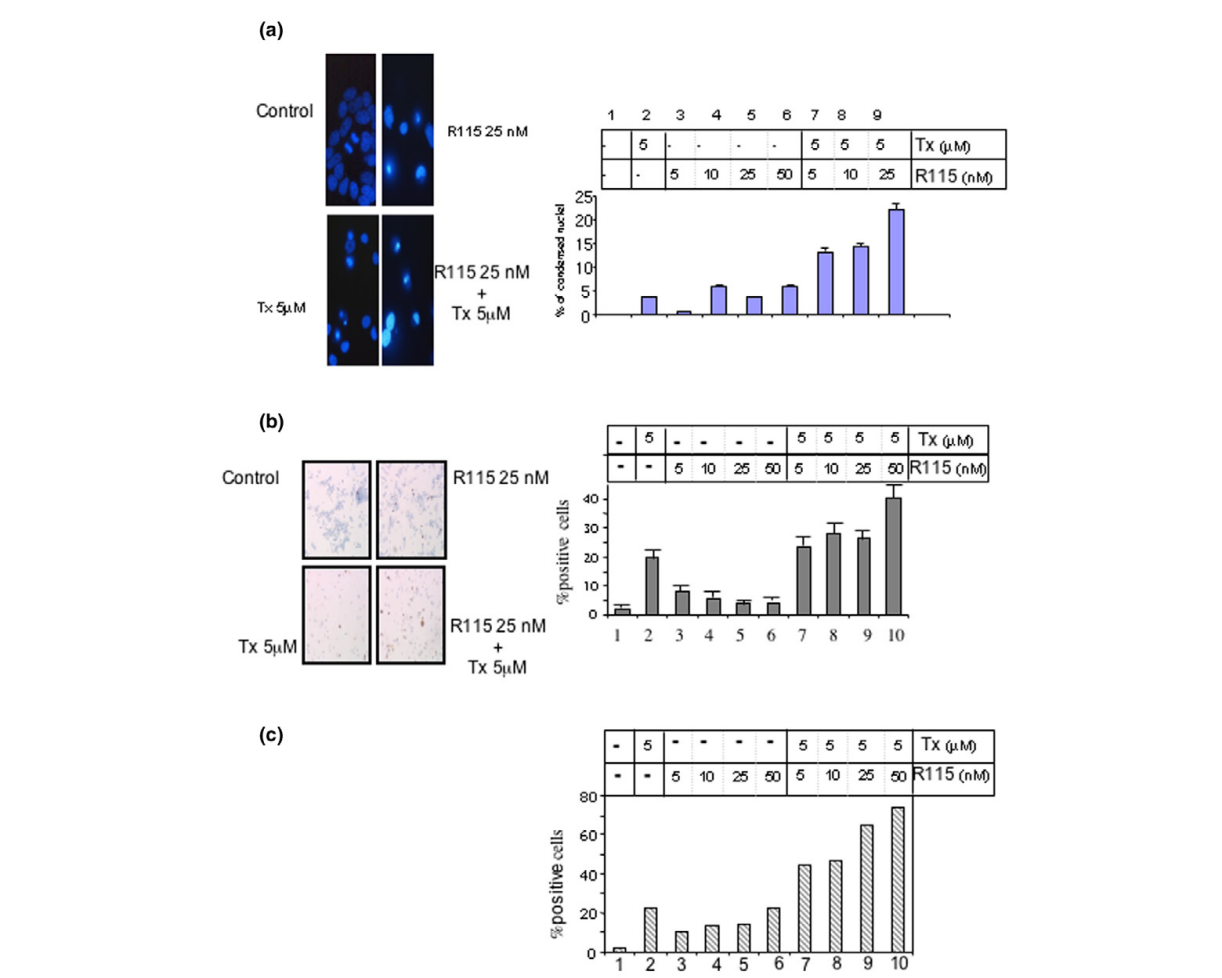
Nuclear condensation of cells incubated for 5 days with tamoxifen (Tx) and R115,777. **(a) **Cells were fixed and stained with the DNA intercalating agent DAPI (left) and nuclei were examined by fluorescence microscopy. DAPI-stained nuclei were counted as normal or condensed nuclei (right). One randomly selected field is presented for each treatment. For three independent experiments, quantification of at least 200 nuclei was performed for each treatment. Error bars indicate the mean values ± standard error of the mean. **(b) **Cells were fixed and stained for the detection of the caspase cleavage product of cytokeratin 18 by immuno-histochemistry. Floating and adherent cells are represented in cytospin preparations. These preparations were fixed and stained with monoclonal antibody M30 CytoDeath. The proportion of positive cells (brown) was calculated as a percentage of the total number of cells. All determinations were performed in triplicate with at least 400 cells being counted. Error bars indicate the mean values ± standard error of the mean. **(c) **Cells were fixed and stained for the detection of the caspase cleavage product of cytokeratin 18 by FACS. Floating and adherent cells were harvested, fixed and stained with the fluoresein-conjugated monoclonal antibody M30 CytoDeath and analysed using flow cytometry. The proportion of positive cells was calculated as a percentage of the total number of cells. Data are representative of one to three independent experiments; intra-assay variations were <1%.

We examined the effects of a combination of Tam and R115,777 on the DNA content of MCF-7 cells using flow cytometry of propidium iodide-stained cells (Fig. 2). The compounds were added for 5 days either individually or in combination and in both cases the appropriate carrier was added as a control. The appearance of a significant fraction of cells with a DNA content less than 2 N would be indicative of apoptotic cells. Such a cell population was not detected among untreated MCF-7 cells. Using these cells we confirmed that Tam treatment induced an increase of sub-G1 DNA content at 1 and 5 μM (Fig. 2, lanes 2, 5, 8 and 11). We also showed that cells incubated with high concentrations of R115,777 (25 or 50 nM) showed an increase of the sub-G1 DNA content equivalent to that obtained with Tam 1 μM. Interestingly, the combination of R115,777 and Tam produced a large increase in the sub-G1 population: 35 ± 5% in the presence of both agents (Fig. 2, lane 13) compared to only 9 ± 3% (Fig. 2, lane 12) or 14 ± 4% (Fig. 2, lane 11) with each agent alone. Cell death was assayed by two other methods to confirm that death detected by flow cytometry of propidium iodide-stained cells was related to apoptosis. First, we used DAPI staining, which determines nuclear morphology (Fig. 3A, left) and counted normal and condensed nuclei (Fig. 3A, right). The number of condensed and fragmented nuclei increased with 5 μM Tam (3.5 ± 0.1%, Fig 3A right lane 2) or with R115,777 (up to 5.9 ± 0.3%, Fig 3A right lanes 3–6) treatment, compared to untreated cells. Once again, the association of Tam (5 μM) with R115,777 (from 5 to 25 nM) significantly enhanced the number of cells with condensed nuclei (13.1 ± 0.6 to 22 ± 1%, Fig 3A right lanes 7–9).

Apoptosis was further assessed using the monoclonal antibody M30 CytoDeath (M30), which is specific for the neo-epitope in cytokeratin 18 that becomes available after an early caspase cleavage during apoptosis. The specific caspase cleavage site within cytokeratin 18 was assessed either immuncytochemically (Fig. 3B) or was analysed by flow cytomoetry (Fig. 3C). We used M30 CytoDeath to selectively stain apoptotic cells, and M30 positive cells were scored (Fig. 3B). Of the cells treated by Tam alone, 19.6% were positive (Fig. 3B, lane 2). This result confirmed that Tam induced cleavage of the specific caspase cleavage site within cytokeratin 18 in breast cancer cells [11,22]. R115,777 treatment induced 4.1% to 8.4% M30 positive cells (Fig 3B, lanes 3–6), as previously reported for other FTIs with mammalian cells [23]. Once again, the combined effects of R115,777 and Tam were higher (up to 40.4%, Fig 3B, lanes 7–10) than that expected from the sum of the individual effects. Flow cytometry analysis confirmed the immunocytochemical counts. R115,777 plus Tam significantly increased the M30-positive cell population (up to 74.2%; Fig. 3C, lanes 7–10). The M30-positive cell population was much smaller following treatment with either R115,777 (10.4% to 22.1%; Fig. 3C, lanes 3–6) or Tam (22.5%; Fig. 3C, lane 2) alone. These results were obtained in three independent experiments with little intra-assay variations; however, the difficulty of cell counting (from 200 to 400 cells per assay) has prevented our carrying out the extensive series of experiments required for isobologram constructions.

Overall, these data show that R115,777, which induces negligible apoptosis by itself, when combined with Tam results in significant apoptosis induction in MCF-7 cells. To differentiate between the involvement of the ER and AEBS pathways in this Tam effect, we compared the apoptosis-inducing activities of ER or AEBS selective ligands

### Effects of R115,777 and different anti-estrogens on caspase cleavage

**Figure 4:**
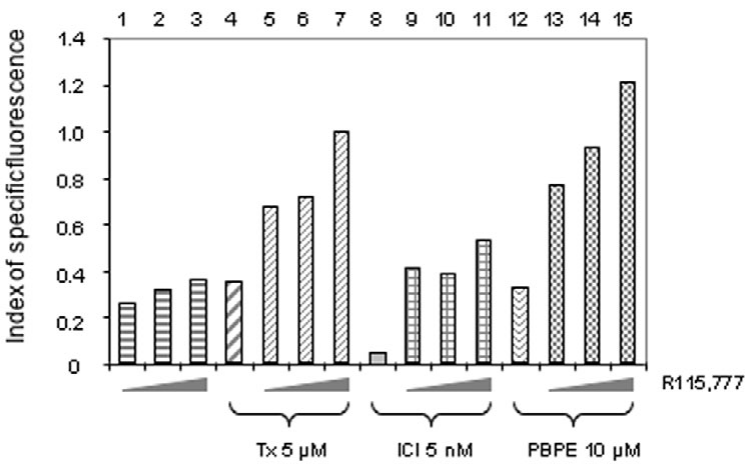
Effects of anti-estrogens and R115,777 on the caspase cleavage product of cytokeratin 18. Determined by FACS on MCF-7 cells incubated for 5 days with either 5 μM tamoxifen (Tx), 1 nM ICI182,780 (ICI) or 10 μM N-pyrrolidine(-phenylmethyl-phenoxy)-ethanamine-HCl (PBPE) combined with 5, 10 or 25 nM R115,777. Floating and adherent cells were harvested, fixed and stained with the fluorescein conjugated monoclonal antibody M30 CytoDeath and analysed using flow cytometry. Results are expressed as an index of specific fluorescence, as described in Materials and methods. Data are representative of one to three independent experiments in duplicate; variations were <1%.

To define any possible involvement of the ER or AEBS pathways in apoptosis induction, a set of experiments similar to those described in Fig. 3c were performed using R115,777 in association with three different 'anti-estrogens', Tam, ICI182,780 or PBPE. Flow cytometric analyses were performed following M30 staining of MCF-7 cells treated for 5 days with these three different combinations of agents. As shown in Fig. 4, 10 μM PBPE was able to increase the number of M30 positive cells (lane 12) to the same extent as 5 μM Tam (lane 4). In contrast, 5 nM ICI182,780 treatment resulted in only modest cytokeratin 18 cleavage (Fig. 4, lane 8). The combination of R115,777 with ICI182,780 resulted in slightly increased M30 staining (Fig. 4, lanes 9–11) compared with R115,777 treatment alone (Fig. 4, lanes 1–3), suggesting that ER is only minimally involved in the synergistic induction of apoptosis resulting from exposure to the combination of R115,777 and Tam.

A similar experiment revealed that the combination of R115,777 with Tam was as efficient in inducing M30 staining (Fig. 4, lanes 5–7) as was R115,777 plus PBPE (Fig. 4, lanes 13–15), suggesting that the major part of the apoptosis inducing effect of Tam either alone or in association with R115,777 is under the control of the AEBS pathway.

These data, therefore, would be consistent with the proposal that the first level of 'cross talk' between the two agents would be that one increases the intracellular concentration of the other, while the second level would be that one binds to the target of the other. To examine these proposals we first used bindings assays and verified that R115,777, like FTI 277, does not interact either with ERs or with AEBS (data not shown).

### R115,777 does not affect the cellular uptake of [^3^H]tamoxifen

We next examined whether R115,777 was able to increase the cellular uptake of Tam. Exposure to increasing concentrations of R115,777 (from 2 to 50 nM) did not modify the cellular level of tritiated Tam in MCF-7 cells (data not shown). Thus, the effects of combining R115,777 with Tam were not attributable to any increase of cellular Tam concentrations by the FTI.

With the unavailability of an assay for R115,777 or a radiolabelled product we were unable to determine the effects of Tam addition on the intracellular concentration of R115,777. To circumvent this problem, we looked for any farnesylation inhibitory activity of R115,777 in the presence or absence tamoxifen.

### Effects of R115,777 and anti-estrogens on isoprenylated proteins

**Figure 5:**
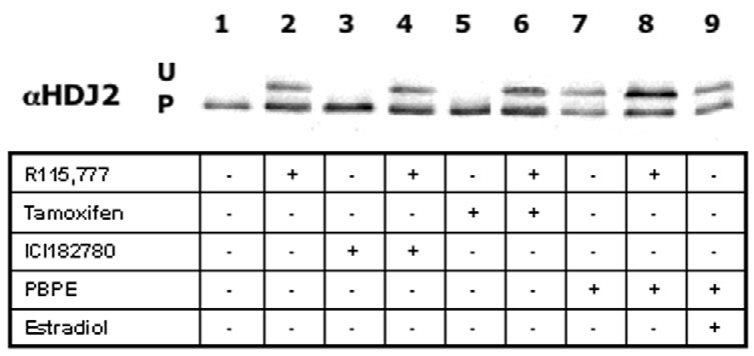
Effects of anti-estrogens and R115,777 on isoprenylated proteins in MCF-7 cells. Cells were incubated for 2 days with R115,777 (1 nM) combined with either tamoxifen (5 μM), ICI182777 (5 nM), N-pyrrolidine(-phenylmethyl-phenoxy)-ethanamine-HCl (PBPE; 10 μM) or estradiol (1 nM). Floating and adherent cell lysates were resolved by SDS-PAGE and blotted onto PVDF membranes and 5 μg protein were loaded for HDJ 2 detection, as described in Materials and methods. U, unprenylated; P, prenylated.

We have previously shown that AEBS is a multiprotein complex consisting of enzymes involved in sterol biosynthesis [8], suggesting that they possibly interfer with the mevalonate pathway. For this reason we examined the capacity of Tam to act on the protein farnesylation process. HDJ2 farnesylation, a surrogate clinical marker for farnesyl transferase inhibitor activity, was used here as a reporter to define any potential inhibitory effect on the mevalonate pathway. Fig. 5 shows western blots of MCF-7 cells incubated with the three 'anti-estrogens'. As a control, untreated cells exhibited complete prenylation of HDJ2 (P, lane 1), while treatment with R115,777 (1 nM) resulted in an accumulation of unprenylated HDJ2, seen as a more slowly migrating band (U, lane 2). Furthermore, addition of neither ICI182,780 (5 nM) nor Tam (5 μM) affected the farnesylation of HDJ2, either alone or in combination with R115,777, which inhibits farnesylation of HDJ2 (lanes 3 to 6), even though the HMG CoA reductase promoter has been shown to be estrogen regulated [24]. The selective AEBS ligand PBPE (10 μM), however, was able to regulate HDJ2 farnesylation itself in the absence of R115,777 (lane 7) and further increased the effect of R115,777 (lane 8). Finally, estradiol did not have any effect, either alone or in association with R115,777, ICI182,780 or Tam (data not shown), or indeed in the presence of PBPE (lane 9).

In this series of experiments, a low 1 nM concentration of R115,777 was used so as to evaluate any increase of the unfarnesylated protein resulting from exposure to the various combinations. Among the three 'anti-estrogens' tested, it is clear that PBPE interferes with the farnesylation of HDJ 2 (Fig. 5), as previously suggested in an earlier study with another AEBS selective ligand in the farnesylation of H-ras [25]. Tam exhibits only a slight or negligible effect on the same protein at concentrations that cause apoptosis and growth inhibition of MCF-7 cells; however, higher concentrations of Tam (50 μM) that induce a high level of cell mortality are able to inhibit HDJ2 prenylation (data not shown). These observations therefore suggest that, although Tam and PBPE bind AEBS with equivalent affinity, they appear to have variable effects on the enzymatic activities of AEBS [5,20].

Any direct or indirect action of the AEBS ligands on the mevalonate pathway were not examined in this study, but the fact that PBPE and Tam did not act similarly on cholesterol biosynthesis [8], inducing the accumulation of different sterols, could be a reason for the differences observed here in their anti-farnesylation activities.

## Conclusion

Although Tam is widely used for the treatment of ER positive breast tumours, we have previously shown that the association of Tam with FTI-277 led to an additive increase in its antiproliferative effects on MCF-7 cells [14]. Furthermore, other authors have reported that hydroxy-tamoxifen (which binds ERs in preference to AEBS) and also ICI182,780 (selective for ER) act synergistically with FTI-277 in inhibiting the growth of ER positive breast tumour cell lines [21]. As 4-hydroxytamoxifen and ICI182,780 have the same ER target, this study was designed to determine which protein targets were involved in the activity of Tam (the clinically used drug) in the presence of a FTI. Bearing in mind that a combination of R115,777 with Tam is already being evaluated in a clinical trial [26,27], we have evaluated the effects of combining one of three 'anti-estrogens' known to have different targets, Tam (ERs and AEBS), ICI182,780 (selective for ERs) and PBPE (selective AEBS ligand), with the FTI R115,777. Whereas both ERs and AEBS appear to be involved in the antiprolliferative effects seen with these combinations, the AEBS pathway seems to be the main target involved in apoptosis induction. Overall, this work reveals that combination of R115,777 with either selective ER ligands or with the selective AEBS ligand are able to induce large increases in their anti-proliferative activities on MCF-7 cells. In view of these distinctive effects, it might be informative to study combinations in different clinical scenarios or employ them in sequence; studying the combination of an FTI with ICI182,780 for early treatment of ER positive breast tumours and reserving combinations with either Tam or a selective AEBS ligand, such as the BMS-217380-01 [28], for more resistant disease.

## Abbreviations

AEBS = anti-estrogen binding site; ER = estrogen receptor; FTI = farnesyl transferase inhibitor; PBPE = N-pyrrolidine(-phenylmethyl-phenoxy)-ethanamine-HCl; PBS = phosphate-buffered saline; SRB = sulphorhodamine B; Tam = tamoxifen.

## Competing interests

The authors declare that they have no competing interests.

## Authors' contributions

The authors' contributions to this work are reflected in the order they are listed above, with the exception of JCF, who supervised the research and the preparation of this report. FD, CM, and MP carried out the experiments and FD drafted the manuscript and performed the statistical analysis. MP provided PBPE and gave helpful advice on its mechanism of action. GF gave helpful advice throughout this study. All authors have read and approved the final manuscript.
